# *In silico *characterization of immunogenic epitopes presented by HLA-Cw*0401

**DOI:** 10.1186/1745-7580-3-7

**Published:** 2007-08-20

**Authors:** Joo Chuan Tong, Zong Hong Zhang, J Thomas August, Vladimir Brusic, Tin Wee Tan, Shoba Ranganathan

**Affiliations:** 1 Institute for Infocomm Research, 21 Heng Mui Keng Terrace, 119613, Singapore; 2 Department of Biochemistry, Yong Loo Lin School of Medicine, National University of Singapore, 8 Medical Drive, 117597, Singapore; 3 Department of Pharmacology and Molecular Sciences, John Hopkins University School of Medicine, Baltimore, MD, USA; 4 Cancer Vaccine Center, Dana-Farber Cancer Institute, Boston, MA, USA; 5 Department of Chemistry and Biomolecular Sciences & Biotechnology Research Institute, Macquarie University, NSW 2109, Australia

## Abstract

**Background:**

HLA-C locus products are poorly understood in part due to their low expression at the cell surface. Recent data indicate that these molecules serve as major restriction elements for human immunodeficiency virus type 1 (HIV-1) cytotoxic T lymphocyte (CTL) epitopes. We report here a structure-based technique for the prediction of peptides binding to Cw*0401. The models were rigorously trained, tested and validated using experimentally verified Cw*0401 binding and non-binding peptides obtained from biochemical studies. A new scoring scheme facilitates the identification of immunological hot spots within antigens, based on the sum of predicted binding energies of the top four binders within a window of 30 amino acids.

**Results:**

High predictivity is achieved when tested on the training (*r*^2 ^= 0.88, *s *= 3.56 kJ/mol, *q*^2 ^= 0.84, *s*_*press *_= 5.18 kJ/mol) and test (A_ROC _= 0.93) datasets. Characterization of the predicted Cw*0401 binding sequences indicate that amino acids at key anchor positions share common physico-chemical properties which correlate well with existing experimental studies.

**Conclusion:**

The analysis of predicted Cw*0401-binding peptides showed that anchor residues may not be restrictive and the Cw*0401 binding pockets may possibly accommodate a wide variety of peptides with common physico-chemical properties. The potential Cw*0401-specific T-cell epitope repertoires for HIV-1 p24^gag ^and gp160^gag ^glycoproteins are well distributed throughout both glycoproteins, with thirteen and nine immunological hot spots for HIV-1 p24^gag ^and gp160^gag ^glycoproteins respectively. These findings provide new insights into HLA-C peptide selectivity, indicating that pre-selection of candidate HLA-C peptides may occur at the TAP level, prior to peptide loading in the endoplasmic reticulum.

## Background

Major histocompatibility complex (MHC) class I molecules, HLA-A, -B, and -C, are cell surface glycoproteins consisting of a polymorphic heavy α chain non-covalently linked to a light chain, β_2_-microglobulin (β_2_m). HLA-A and -B molecules play critical roles in cell mediated immune responses by binding short antigenic peptide fragments and presenting them on the surface of antigen-presenting cells for recognition by the CD8^+ ^cytotoxic T lymphocyte (CTL). Although several HLA-C specificities with CTL epitopes have been reported [1,2], much remains unknown with regards to their role in the immune response against viral antigens in part due to their poor expression at the cell surface [3,4]. Recent research shows that this group of molecules plays a major role in the control of human immunodeficiency virus type 1 (HIV-1) infection [5]. Improved understanding of peptide binding to this group of molecules is important in the study of HIV-1 disease progression, as well as the design of effective HIV peptide vaccines.

The HLA-C allele, Cw*0401, is of particular interest in the study of HIV-1 disease progression because it is the restriction element for HIV-1 proteins [5]. Two HIV-1 proteins (p24^gag ^and gp160^gag^) are currently known to be restricted by Cw*0401 [5]. Cw*0401 is present in approximately 10% of the general population [6]. The allele is expressed intracellularly in amounts comparable with HLA-A and -B molecules, but is poorly expressed at the cell surface [7,8]. Improved understanding of peptide binding to this molecule is important for elucidating its role in HIV-1 disease progression.

Computational strategies for prediction of peptide binding to HLA-A and -B molecules are relatively advanced [9], while sequence-based predictive models for HLA-C molecules have encountered limited success due to the lack of experimental training data [10]. Two matrix-based prediction algorithms for Cw*0401 were reported [11,12], but a sequence independent approach is still lacking. To overcome these limitations, we have developed a structure-based predictive technique that integrates the strength of Monte Carlo simulations and homology modeling [13-15]. This method utilizes a probe or "base fragment" to sample different regions of the receptor binding site, followed by loop closure and refinement of the entire class I peptide. The technique has been successfully applied to analyze peptides binding to a variety of MHC class II alleles [14,15]. In this work, we now extend our analysis to peptides presented by the class I HLA-C molecule. We investigated the HIV-1 p24^gag ^and gp160^gag ^peptide binding repertoire of Cw*0401 and illustrate that areas with high concentration of T-cell epitopes or "immunological hot spots" are potentially well distributed throughout both HIV-1 p24^gag ^and gp160^gag^. We also show that Cw*0401 can possibly bind antigenic peptides in amounts comparable to both HLA-A and -B molecules. Characterization of predicted Cw*0401 binding sequences reveal that Cw*0401 may bind a large variety of amino acids at anchor positions with common physico-chemical properties which correlate well with existing experimental studies [11].

## Results and discussion

### Cw*0401 predictive model

High predictivity (*r*^2 ^= 0.88, *s *= 3.56 kJ/mol, q^2 ^= 0.84, s_press _= 5.18 kJ/mol) is achieved when tested on the training dataset of 6 Cw*0401 peptide sequences. The Cw*0401 predictive model outperforms the predictive models done by Rognan *et al*. [16] on training datasets of 5 A*0204 (*r*^2 ^= 0.85, *s*_*press *_= 2.40 kJ/mol) and 37 2K^k ^(*r*^2 ^= 0.78, *s*_*press *_= 3.16 kJ/mol) peptide sequences and is comparable with our previous DRB1*0402 (r^2 ^= 0.90, s = 1.20 kJ/mol, q^2 ^= 0.82, s_press _= 1.61 kJ/mol) and DQB1*0503 (r^2 ^= 0.95, s = 1.20 kJ/mol, q^2 ^= 0.75, s_press _= 2.15 kJ/mol) prediction models on a training set of 8 peptides [17]. The cross-validation coefficient *q*^2 ^and the standard error of prediction *s*_*press *_are stable, with *q*^2 ^= 0.84 and *s*_*press *_= 5.18 kJ/mol. This iterative regression procedure validates the internal consistency of the scoring function in the current model, rendering it suitable for predictions on the test dataset obtained from biochemical studies. The predictive performance of our model is further validated using the test dataset of 58 peptides. The external validation results indicate that our Cw*0401 predictive model is suitable for discriminating binding ligands from the background with high accuracy (A_ROC _= 0.93) with sensitivity of 76% (SP = 0.95).

### Characterization of Cw*0401 binding peptides

An in-depth analysis was performed to investigate the characteristics of Cw*0401 binding peptides. A panel of 2279 sequences was generated using an overlapping sliding window of size 9 across the entire p24^gag ^[5] and gp160^gag ^[18] glycoproteins and modeled into the binding groove of Cw*0401. From these sequences, a total of 877 binding sequences (predicted binders; SE = 76%, SP = 80%) were selected and a systematic analysis was performed to analyze the number of occurrence of individual amino acid residues and physico-chemical properties [19] at each position of Cw*0401 binding peptides.

Peptide position p2 is characterized by alanine (10%), glycine (13%), leucine (9%), serine (9%). 60% of the predicted residues at this position are hydrophobic in nature, while 93% are neutral. These properties correlate with the physico-chemical properties of existing binding motif (Tyr/Phe) at p2 [11] as well as with the observed conservation in the test data (Table 1: Phe – 58% and Tyr – 26%). The p3 position shows a strong preference for glycine (11%) and threonine (8%). Existing anchor residues at this position are aspartic acid and histidine, which accounts for 9% of the total position-specific composition in the dataset. The p4 position shares similar characteristics as p3 (glycine: 9%; threonine: 8%), with additional preference for leucine (8%). Similar results were obtained at the p5 position (alanine: 8%; glycine: 9%; leucine: 8%). At p6, characteristic residues include glycine (9%) and leucine (8%). This position is favored by neutral (81%) acyclic (89%), medium/large (77%), and hydrophobic (51%) residues. The physico-chemical properties of these residues are in agreement with Val/Ile/Leu as reported in earlier studies [11] and is comparable with the conservation in the test dataset reported in Table 1 (Val – 29%, Ile – 12%, Leu and Pro – 10%). Finally, the p9 position was defined by six amino acids, including alanine (8%), glycine (10%), isoleucine (8%), leucine (8%), threonine (9%), and valine (8%). This position is primarily dominated by neutral (90%) and hydrophobic (58%) residues, and agrees with profiles of Leu/Phe as previously reported [11] and is consistent with the conservation in the test dataset reported in Table 1 (Leu and Phe – 39%,). Collectively, our data indicates that individual binding pockets may not be highly specific as previously reported [11] but can rather accommodate a wide-range of anchor residues with common physico-chemical properties. We attribute the discrepancies to two possibilities: i) the lack of extensive research on Cw*0401 peptides; and ii) natural peptides carrying these residues may be present in small amount and were thus not detected by experimental studies.

**Table 1:** HLA-Cw4 dataset used in this study.

**No.**	**Category**	**Source**	**Peptide**	**IC_50 _(nM)**	**Ref.**
1	Training Set	Cw3 consensus	FAMPNFQTL	651	24
2	Training Set	Cw6 consensus	IPFPIVRYL	>30000	24
3	Training Set	Cw7 consensus	KYPDFVDAL	2.4	24
4	Training Set	Cw4 consensus	QYDDAVYKL	18	24
5	Training Set	Histone H3.3	RYRPGTVAL	>30000	24
6	Training Set	Unknown Cw6 natural ligand	YQFTGIKKY	>30000	24
7	Test Set	Transcripton factor SUPT4H 56–64	SFDGIIAMM	Binder	10
8	Test Set	Transducin-like 3 120–128	AFDPTSTLL	Binder	10
9	Test Set	UBE3B variant 1 742–750	VFDPALNLF	Binder	10
10	Test Set	XP_173235 3–11	LFDITGQDF	Binder	10
11	Test Set	8 59–67	VYDTNPAKF	Binder	10
12	Test Set	Acyl-CoA synthetase 4 82–90	LFDHAVSKF	Binder	10
13	Test Set	Adenosylhomocyteinehydrolase 566–574	SFDAHLTEL	Binder	10
14	Test Set	ART-1 Adenocarcinoma antigen 21–29	SFDLLPREF	Binder	10
15	Test Set	ATP-binding cassette, sub-family F, member 3 500–508	YYDPKHVIF	Binder	10
16	Test Set	Block of proliferation 1 639–647	SYDSKLVWF	Binder	10
17	Test Set	BM-015 144–152	HFDPEVVQI	Binder	10
18	Test Set	CDC45 541–549	HFDLSVIEL	Binder	10
19	Test Set	Cholesterol acyltransferase 72–80	HFDDFVTNL	Binder	10
20	Test Set	Chromosome 20 open reading frame 40 318–326	FFDNISSEL	Binder	10
21	Test Set	Elongation factor 2 265–273	YFDPANGKF	Binder	10
22	Test Set	Epithelial cell transforming oncogene 21–29	IFDSKVTEI	Binder	10
23	Test Set	Ethanolamine kinase EKI1 132–141	HWDPQEVTL	Binder	10
24	Test Set	Eukaryotic translation initiation factor 3, Su 6 interacting protein 478–486	FLDLTEGEF	Binder	10
25	Test Set	Fatty acid synthetase 544–552	TFDDIVHSF	Binder	10
26	Test Set	FK506 binding protein 9 303–311	VFDIHVIDF	Binder	10
27	Test Set	Glutamine:fructose-6-phosphate amidotransferase (GFAT) 345–353	NFDDYTVNL	Binder	10
28	Test Set	Tousled-like kinase 456–464	AFDLTEQRY	Binder	10
29	Test Set	Transcription factor IIE 144–152	LFDPMTGTF	Binder	10
30	Test Set	HSP 70 kDa 1A 179–205	IFDLGGGTF	Binder	10
31	Test Set	HSPC198 19–27	SYDLFVNSF	Binder	10
32	Test Set	HSP J2 156–164	SFDTGFTSF	Binder	10
33	Test Set	Hypothetical protein FLJ00365 80–88	YFDAIPVTM	Binder	10
34	Test Set	Hypothetical protein FLJ11220 373–381	YLPDFLDYF	Binder	10
35	Test Set	Hypothetical protein FLJ20343 415–423	RFDEAYIYM	Binder	10
36	Test Set	Insuline degrading enzyme IDE 150–158	YFDVSHEHL	Binder	10
37	Test Set	Integrin alpha-V (Vitronectin) 224–232	KYDPNVYSI	Binder	10
38	Test Set	KCIP-1 186–194	AFDEAIAEL	Binder	10
39	Test Set	KIAA0461 721–729	SMDPLPVFL	Binder	10
40	Test Set	KIAA1463 444–452	FYDERIVVV	Binder	10
41	Test Set	KIAA1921 350–358	VYDGKIYTL	Binder	10
42	Test Set	Metalloproteinase 10 331–339	FWPSLPSYL	Binder	10
43	Test Set	Methionine-tRNA synthetase 2 (mitochondrial) 58–66	YYDEKVVKL	Binder	10
44	Test Set	Mucin-5B 168–176	TFDGTSYTF	Binder	10
45	Test Set	Myosin phosphatase target subunit 1 37–45	KFDDGAVFL	Binder	10
46	Test Set	Nuclear autoantigenic sperm protein 599–607	QYDEAVAQF	Binder	10
47	Test Set	Nucleoporin NUP358 261–269	SFDSALQSV	Binder	10
48	Test Set	P621 70–78	VFDKTLAEL	Binder	10
49	Test Set	Phosphate carrier precursor 331–339	IYDSVKVYF	Binder	10
50	Test Set	PRO2242 61–69	YFDPQYFEF	Binder	10
51	Test Set	Protein phosphatase 6 102–110	KWPDRITLL	Binder	10
52	Test Set	Putative prostate tumor suppressor 165–173	TFDLQRIGF	Binder	10
53	Test Set	Rac1 168–176	VFDEAIRAV	Binder	10
54	Test Set	RNA Helicase A 697–705	VFDPVPVGV	Binder	10
55	Test Set	Similiar to KIAA1911 80–88	FWDGKIVLV	Binder	10
56	Test Set	Topoisomerase I 241–249	YYDGKVMKL	Binder	10
57	Test Set	Tensin 3 143–151	FYDDKVSAL	Binder	10
58	Test Set	HIV-1 (BRU) gp120 350–358	SFNCGGEFF	Binder	18
59	Test Set	HIV-1 (BRU) gag p24 307–315	QASQEVKNW	Binder	21
60	Test Set	Synthetic peptide	AYDDAVYKL	Binder	25
61	Test Set	Cw7 consensus	AYADFVYAY	>30000	24
62	Test Set	CKShs2	KYFDEHYEY	>30000	24
63	Test Set	Unknown Cw6 natural ligand	YRHDGGNVL	>30000	24
64	Test Set	Unknown Cw7 natural ligand	NKADVILKY	>30000	24

### Prediction of p24^gag ^and gp160^gag ^immunological hot spots

**Figure 1:**
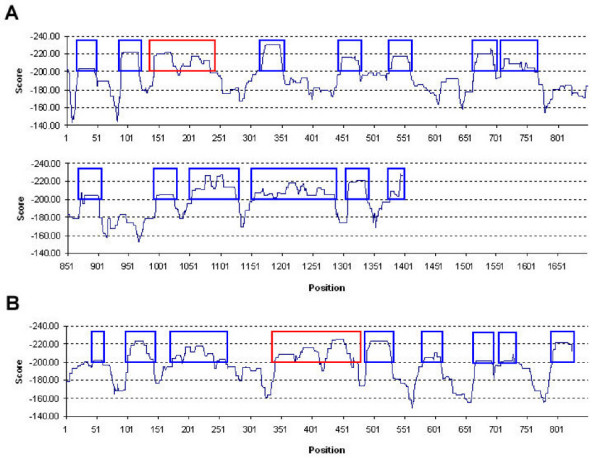
Predicted start positions of Cw*0401-specific hotspots (sliding window size = 30) along (A) p24^gag ^and (B) gp160^gag ^glycoproteins. Scores are computed based on the sum of predicted binding energies of top four binders within the 30 amino acid sliding window. Predicted hotspots regions are shown in blue boxes with experimentally verified regions shown in red.

The potential T-cell epitope repertoire (data not shown) and immunological hot spots (Figure 1) for HIV-1 p24^gag ^and gp160^gag ^are well distributed throughout both glycoproteins. The known p24^gag ^epitope (p24 168–175) and gp160^gag ^epitope (gp160 375–383) were successfully predicted by our model at the threshold of -200 (SE = 76%, SP = 80%) [5,20]. At this threshold, the number of predicted hot spots for p24^gag ^are fourteen (p24 20–75, 89–148, 143–262, 311–381, 447–506, 526–590, 664–727, 708–791, 873–930, 996–1053, 1054–1157, 1157–1316, 1308–1368, and 1378–1427), with an estimated FN = 3, and FP = 3 for SE = 0.76 and SP = 0.95 (Figure 1 and Table 2). For gp160^gag ^we predict nine hot spots (gp160 44–89, 102–171, 174–289, 341–504, 489–559, 581–639, 667–721, 708–766, and 793–852), with an estimated FN = 2, FP = 2 for SE = 0.76 and SP = 0.95 (Figure 1 and Table 3). The results presented here indicate that Cw*0401 can bind antigenic peptides with specificities comparable to HLA-A and -B molecules, and any variability in antigen expression may be directly related to the loss or reduced cell surface expression of the molecule by mechanisms as yet unknown [8,21].

**Table 2:** Predicted Cw*0401-specific immunological hotspots for p24^gag^.

No.	Position	Sequence
1	20–75	RLRPGGKKKYKLKHIVWASRELERFAVNPGLLETSEGCRQILGQLQPSLQTGSE EL
2	89–148	YNTVATLYCVHQRIEIKDTKEALDKIEEEQNKSKKKAQQAAADTGHSNQVSQNY PIVQNIQGQMVHQAIS
3	143–262	PIVQNIQGQMVHQAISPRTLNAWVKVVEEKAFSPEVIPMFSALSEGATPQDLNT MLNTVGGHQAAMQMLKETINEEAAEWDRVHPVHAGPIAPGQMREPRGSDIAGTT STLQEQIGWMTNNPPIPVGEIY
4	311–381	QEVKNWMTETLLVQNANPDCKTILKALGPAATLEEMMTACQGVGGPGHKARVLA EAMSQVTNSATIMMQRG
5	447–506	EFSSEQTRANSPTRRELQVWGRDNNSPSEAGADRQGTVSFNFPQVTLWQRPLVT IKIGGQ
6	526–590	LPGRWKPKMIGGIGGFIKVRQYDQILIEICGHKAIGTVLVGPTPVNIIGRNLLT QIGCTLNFPIS
7	664–727	FRELNKRTQDFWEVQLGIPHPAGLKKKKSVTVLDVGDAYFSVPLDEDFRKYTAF TIPSINNETP
8	708–791	DEDFRKYTAFTIPSINNETPGIRYQYNVLPQGWKGSPAIFQSSMTKILEPFRKQ NPDIVIYQYMDDLYVGSDLEIGQHRTKIEE
9	873–930	TKALTEVIPLTEEAELELAENREILKEPVHGVYYDPSKDLIAEIQKQGQGQWTY QIYQ
10	996–1053	TWIPEWEFVNTPPLVKLWYQLEKEPIVGAETFYVDGAANRETKLGKAGYVTNRG RQKV
11	1054–1157	VTLTDTTNQKTELQAIYLALQDSGLEVNIVTDSQYALGIIQAQPDQSESELVNQ IIEQLIKKEKVYLAWVPAHKGIGGNEQVDKLVSAGIRKVLFLDGIDKAQDE
12	1157–1316	DEHEKYHSNWRAMASDFNLPPVVAKEIVASCDKCQLKGEAMHGQVDCSPGIWQL DCTHLEGKVILVAVHVASGYIEAEVIPAETGQETAYFLLKLAGRWPVKTIHTDN GSNFTGATVRAACWWAGIKQEFGIPYNPQSQGVVESMNKELKKIIGQVRDQA
13	1308–1368	IIGQVRDQAEHLKTAVQMAVFIHNFKRKGGIGGYSAGERIVDIIATDIQTKELQ KQITKIQ
14	1378–1427	RNPLWKGPAKLLWKGEGAVVIQDNSDIKVVPRRKAKIIRDYGKQMAGDDC

**Table 3:** Predicted Cw*0401-specific immunological hotspots for gp160^gag^.

No.	Position	Sequence
1	44–89	VWKEATTTLFCASDAKAYDTEVHNVWATHACVPTDPNPQEVVLVNV
2	102–171	EQMHEDIISLWDQSLKPCVKLTPLCVSLKCTDLGNATNTNSSNTNSSSGEMM MEKGEIKNCSFNISTSIR
3	174–289	VQKEYAFFYKLDIIPIDNDTTSYTLTSCNTSVITQACPKVSFEPIPIHYCAP AGFAILKCNNKTFNGTGPCTNVSTVQCTHGIRPVVSTQLLLNGSLAEEEVVI RSANFTDNAKTI
4	341–504	AKWNATLKQIASKLREQFGNNKTIIFKQSSGGDPEIVTHSFNCGGEFFYCNS TQLFNSTWFNSTWSTEGSNNTEGSDTITLPCRIKQFINMWQEVGKAMYAPPI SGQIRCSSNITGLLLTRDGGNNNNGSEIFRPGGGDMRDNWRSELYKYKVVKI EPLGVAPT
5	489–559	KYKVVKIEPLGVAPTKAKRRVVQREKRAVGIGALFLGFLGAAGSTMGARSMT LTVQARQLLSGIVQQQNN
6	581–639	LQARILAVERYLKDQQLLGIWGCSGKLICTTAVPWNASWSNKSLEQIWNNMT WMEWDRE
7	667–721	ELDKWASLWNWFNITNWLWYIKIFIMIVGGLVGLRIVFAVLSIVNRVRQGYS PLS
8	708–766	SIVNRVRQGYSPLSFQTHLPTPRGPDRPEGIEEEGGERDRDRSIRLVNGSLA LIWDDLR
9	793–852	RGWEALKYWWNLLQYWSQELKNSAVSLLNATAIAVAEGTDRVIEVVQGACRA IRHIPRRI

## Conclusion

Due to the low expression of HLA-C molecules at the cell surface, their role in cell mediated immune responses remain poorly understood. Collectively, the outcome of this analysis provides insights into the binding specificities of Cw*0401. Our data strongly indicate that Cw*0401 can bind antigenic peptides in amounts comparable to both HLA-A and -B molecules, and show the existence of a potentially large number of Cw*0401-specific T-cell epitopes that are evenly distributed throughout both HIV-1 p24^gag ^and gp160^gag ^glycoproteins. It remains to be determined what proportion of these peptides may be expressed at the cell surface and capable of eliciting functional responses. Probably, pre-selection of candidate HLA-C peptides may occur at the TAP level, prior to peptide loading in the ER [8]. Consequently, a higher concentration of peptides is necessary for complexation with HLA-C molecules, resulting in their release from TAP. This provides a possible explanation for the reduced cell surface expression of HLA-C molecules [8].

## Methods

### Data

#### Crystallographic data

The coordinates of Cw*0401 were obtained from the Protein Databank (PDB) with PDB code 1QQD [22]. The structure was relaxed by conjugate gradient minimization, using the Internal Coordinate Mechanics (ICM) software [23].

#### Experimental binding data

The dataset comprises a total of 64 (57 binders and 7 non-binders) 9-mer peptides (Table 1). The available dataset is divided into training and testing datasets. Peptides with experimental IC_50 _values were selected as training data for optimizing the empirical free energy function (refer *Empirical Free Energy Function*). Due to the lack of experimental data, only 9 peptides with experimental IC_50 _values were identified, six (three binders and three non-binders) of which were used for training, while the remainder (four non-binders) were included in the test dataset as true negatives. Therefore, the training dataset contained six peptides with experimentally determined IC_50 _values (2 high-affinity binders, 1 medium-affinity binder, and 3 non-binders) derived from biochemical studies [24], while the testing dataset comprised the remainder 58 peptides (54 binders and 4 non-binders) [10,18,21,24,25]. Experimental IC_50 _values were classified as follows – high-affinity binders: IC_50 _≤ 500 nM, medium-affinity binders: 500 nM < IC_50 _≤ 1500 nM, low-affinity binders: 1500 < IC_50 _≤ 5000 nM and non-binders: 5000 < IC_50_.

#### HIV-1 sequence data

The sequences of HIV-1 p24^gag ^and gp160^gag ^glycoproteins were obtained from UniProt [26]. The accession numbers for p24^gag ^and gp160^gag ^glycoproteins used in this study are P04585 and P03377 respectively.

### Model

#### Peptide docking

Docking was performed according to the procedure utilized in previous similar works [13-15]: (i) pseudo-Brownian rigid body docking of peptide fragments to the ends of the binding groove, (ii) central loop closure by satisfaction of spatial constraints, and (iii) refinement of the backbone and side-chain atoms of the ligand and receptor contact regions.

#### Empirical free energy function

The scoring function presented herein is based on the free energy potential in ICM [23]. Computation of the binding free energy was performed according to previous similar work based on the difference between the energy of the solvated complex and the sum of the energy of the solvated receptor and that of the peptide ligand, followed by optimization using experimental IC_50 _values [15]. This step was followed by 6-fold cross-validation for assessment of quality of the scoring function [15]. In *k*-fold cross-validation, *k *random, (approximately) equal-sized, disjoint partitions of the sample data were constructed, and all given models were trained on (*k*-1) partitions and tested on the excluded partition. The results were averaged after *k *such experiments, and thus the observed error rate may be taken as an estimate of the error rate expected upon generalization to new data. The predictive power of the models was assessed by the cross-validation coefficient *q*^2 ^and the standard error of prediction *s*_*press*_.

#### Immunological hot spot prediction

In this study, 'immunological hot spots' are defined as antigenic regions of up to 30 amino acids and modeled according to previous similar work based on the sum of predicted binding energies of the top four binders within a window of 30 amino acids [27]. Where available, these predicted hotspots were validated with available experimentally determined sites.

### Training, testing and validation

The free energy scoring function was calibrated using 6 peptides with experimental IC_50 _values and tested on a dataset 58 peptides (54 binders and 4 non-binders) obtained from biochemical studies (Table 1). The predictive performance of our model was assessed using sensitivity (SE), specificity (SP) and receiver operating characteristic (ROC) analysis [28]. SE = TP/(TP+FN) and SP = TN/(TN+FP), indicate percentages of correctly predicted binders and non-binders, respectively. TP (true positives) represents correctly predicted experimental binders and TN (true negatives) for experimental non-binders incorrectly predicted as binders. FN (false negatives) denotes experimental binders predicted as non-binders and FP (false positives) stands for experimental non-binders predicted as binders. The accuracy of our predictions was assessed by the ROC analysis where the ROC curve is generated by plotting SE as a function of (1-SP) for a complete range of classification thresholds. The area under the ROC curve (A_ROC_) provides a measure of overall prediction accuracy, A_ROC _< 70% for poor, A_ROC _> 80% for good and A_ROC _> 90% for excellent predictions [15].

## Abbreviations

CTL, cytotoxic T lymphocyte; HLA, human leukocyte antigen; MHC, major histocompatibility complex; ER, endoplasmic reticulum; HIV, human immunodeficiency virus; ROC, receiver operating characteristic; SE, sensitivity; SP, specificity; TN, true negative; TP, true positive; FN, false negative; FP, false positive

## Authors' contributions

JCT carried out the computational modeling studies, participated in data analysis and drafted the manuscript. ZHZ helped in data collection and computational modeling studies. JTA, TWT, VB participated in experimental design. SR conceived the study, and participated in its design and coordination and finalized the manuscript. All authors read and approved the final manuscript.
